# Fluorescence angiography likely protects against anastomotic leak in colorectal surgery: a systematic review and meta-analysis of randomised controlled trials

**DOI:** 10.1007/s00464-022-09255-1

**Published:** 2022-05-04

**Authors:** Jeremy Meyer, Heman Joshi, Nicolas C. Buchs, Frédéric Ris, Justin Davies

**Affiliations:** 1 Cambridge Colorectal Unit, Addenbrooke’s Hospital, Cambridge University Hospitals NHS Foundation Trust, Cambridge, CB2 0QQ UK; 2 Division of Digestive Surgery, University Hospitals of Geneva, Rue Gabrielle-Perret-Gentil 4, 1211 Genève 14, Switzerland; 3 Medical School, University of Geneva, Rue Michel-Servet 1, 1205 Genève, Switzerland; 4 University of Cambridge, Cambridge, UK

**Keywords:** Angiography, Anastomotic leak, Bowel division, Ischaemia, Prevention, Colorectal surgery, Colectomy

## Abstract

**Objective:**

Observational studies have shown that fluorescence angiography (FA) decreases the incidence of anastomotic leak (AL) in colorectal surgery, but high-quality pooled evidence was lacking. Therefore, we aimed at confirming this preliminary finding using a systematic review and meta-analysis of randomised controlled trials (RCTs) in the field.

**Methods:**

MEDLINE, Embase and CENTRAL were searched for RCTs assessing the effect of intra-operative FA versus standard assessment of bowel perfusion on the incidence of AL of colorectal anastomosis. The systematic review complied with the PRISMA 2020 and AMSTAR2 recommendations and was registered in PROSPERO. Pooled relative risk (RR) and pooled risk difference (RD) were obtained using models with random effects. Heterogeneity was assessed using the Q-test and quantified using the I^2^ value. Certainty of evidence was assessed using the GRADE Pro tool.

**Results:**

One hundred and eleven articles were screened, 108 were excluded and three were kept for inclusion. The three included RCTs compared assessment of the perfusion of the bowel during creation of a colorectal anastomosis using FA versus standard practice. In meta-analysis, FA was significantly protective against AL (3 RCTs, 964 patients, RR: 0.67, 95% CI: 0.46 to 0.99, I^2^: 0%, *p* = 0.04). The RD of AL was non-significantly decreased by 4 percentage points (95%CI: − 0.08 to 0, I^2^: 8%, *p* = 0.06) when using FA. Certainty of evidence was considered as moderate.

**Conclusion:**

The effect of FA on prevention of AL in colorectal surgery exists but is potentially of small magnitude. Considering the potential magnitude of effect of FA, we advise that future RCTs have an adequate sample size, include a cost-benefit analysis of the technique and better define the subpopulation who could benefit from FA.

**Graphical abstract:**

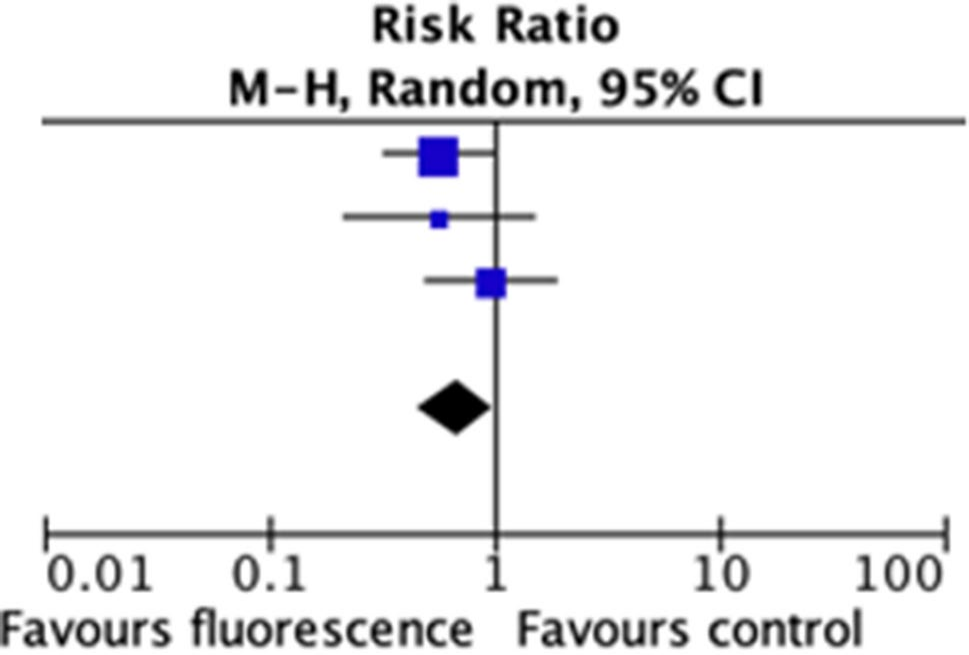

**Supplementary Information:**

The online version contains supplementary material available at 10.1007/s00464-022-09255-1.

Anastomotic leak (AL) is one of the most feared complications in colorectal surgery and occurs in up to 8.1% of patients after right hemicolectomy according to the European Society for Coloproctology snapshot audit [1] and potentially up to 17.1% after low anterior resection according to the GRECCAR 5 trial [2]. Several pre-operative, peri-operative and post-operative risk factors for AL have been identified [3, 4] and have been the subjects of targeted interventions aiming at reducing the incidence of AL. Among these factors, poor perfusion of the two bowel ends was identified as a cause of AL which could be avoided, and numerous methods allowing intra-operative assessment of bowel perfusion and to aid the surgeon in defining the level of bowel division have been developed over the years [5]. Recently, intra-operative fluorescence angiography (FA) has gained tremendous interest and has been the subject of numerous publications showing encouraging results [6–13]. Of note, the PILLAR II study showed that FA led to a change in the level of bowel division in 7.9% of patients and may have avoided potential AL in these patients [14]. Systematic reviews and meta-analyses have demonstrated that FA allowed reductions in the incidence of AL [7–9], of reoperations [7, 8] and of complications [7, 8] in colorectal surgery. The beneficial effect on AL was observed on AL of grades A, B and C [9].

However, high-quality evidence has been lacking in terms of reaching definitive conclusions. Of note, the first RCT in the field by De Nardi et al. did not report any significant advantage of FA over macroscopic observation of the bowel in terms of incidence of AL [15]. Considering the importance of preventing AL for patients and healthcare systems, as well as the release of two new RCTs [16, 17], we aimed to pool the most recent published high-quality evidence on the subject.

## Materials and methods

The work was reported in line with the PRISMA 2020 (Preferred Reporting Items for Systematic Reviews and Meta-Analyses) [18] and AMSTAR 2 (Assessing the methodological quality of systematic reviews) guidelines. MEDLINE, Embase and CENTRAL were searched without time limit to the 13.07.2021 for RCTs written in English and assessing the effect of intra-operative FA *versus* standard assessment of bowel perfusion on the incidence of AL after colorectal surgery anastomosis (Table S1). Observational and/or non-randomised studies, letters, congress abstracts and secondary analyses were excluded. Publications not reporting on the incidence of AL in the group with FA and in the group without FA were excluded. Two independent reviewers (JM, HJ) performed the screening of eligible articles and the data extraction. In case of disagreement, consensus was reached with a third reviewer (RJD). Pooled relative risk (RR) and pooled risk difference (RD) were obtained using models with random effects. The number needed to treat (NNT) was calculated as 1/(-RD). Heterogeneity was assessed using the Q-test and quantified using the *I*^2^ value. Risk of bias was assessed using the Cochrane Collaboration’s tool 2 for assessing risk of bias [19]. The software Review Manager (RevMan 5, version 5.3, Copenhagen: the Nordic Cochrane Centre, 2014) was used for meta-analysis. The software GRADE PRO [20] was used for GRADE assessment of the certainty of evidence. Institutional review board approval was not required. The systematic review protocol was registered in PROSPERO (CRD42021272139).

## Results

### Inclusion process

One hundred and eleven articles were screened, 108 were excluded and three [15–17] were included in the qualitative and quantitative analyses (Figure S1).

### Characteristics of included studies

The three included RCTs [15–17] compared assessment of the perfusion of the bowel during creation of a colorectal anastomosis using FA *versus* standard practice Publications notably included the FLAG trial [16] and the recent PILLAR III trial [17]. One publication was from Russia [16], one from Italy [15] and one from the USA [17]. Two trials were multicentre [15, 17] and one was from a single centre [16]. The number of included participants ranged from 240 [15] to 377 [16]. The PILLAR III trial had to be stopped before reaching its expected sample size (due to recruitment below expectations) and included 247 patients [17]. Two RCTs included patients who underwent elective stapled colorectal anastomosis for benign or malignant diseases [15, 16], and one RCT only included patients with malignant disease [17]. Interventions performed were left colectomy or anterior resection with partial or total mesorectum excision, using a minimally invasive approach from half of the patients [16] to more than 80% of the patients [17]. Colorectal anastomoses were created less than 15 cm from the anal verge [16], less than 10 cm from the anal verge [17] and between 2 and 15 cm from the anal verge [15]. Defunctionning loop ileostomy was created in 71.1% of patients in the FA group, and in 70.5% of patients in the control group in the RCT by Alekseev et al. [16]; and in 73.7% of patients in the FA group and 80.4% of those in the control group in the PILLAR III trial [17]. Incidence of AL was measured within 30 days [16] or within 8 weeks [17] after the surgical procedure, and was determined following investigations in a symptomatic patient and/or using contrast examination of the colorectal anastomosis [16], and contrast examination and/or endoscopy in patients with loop ileostomy [17]. Summary details of included RCTs are reported in Table 1. Summary details of FA interventions are reported in Table S2.

**Table 1:** Characteristics of included studies

Authors	Year	Country	Acronym	Period	Patients, n	Population	Intervention	Control	Outcome
Alekseev et al. [16]	2020	Russia	FLAG	11.2017–08.2019	377	Elective stapled colorectal anastomosis located below 15 cm from the anal verge for benign or malignant disease	ICG fluorescence angiography	Macroscopic appearance of the bowel	Anastomotic leak < 30 days
De Nardi et al. [15]	2020	Italy	–	01.2016–11.2017	240	Elective stapled colorectal anastomosis located 2-15 cm from the anal verge for benign or malignant disease	ICG fluorescence angiography	Macroscopic appearance of the bowel + perfusion of marginal blood vessels	Anastomotic leak < 30 days
Jafari et al. [14]	2021	USA	PILLAR III	03.2015–02.2017	347	Elective stapled colorectal anastomosis located up to 10 cm from the anal verge for malignant disease	ICG fluorescence angiography	No fluorescence angiography	Anastomotic leak < 8 weeks

### Effect of fluorescence angiography on anastomotic leak

**Fig. 1:**
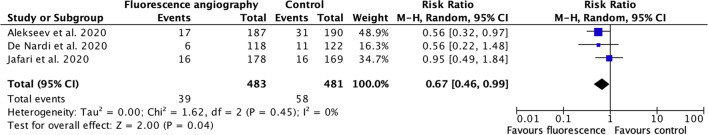
Meta-analysis of fluorescence angiography versus control for anastomotic leak. Forest plot comparing fluorescence angiography versus control for anastomotic leak. Each horizontal bar summarises a study. The bars represent 95% confidence intervals. The squares inform on each of the studies’ weight in the meta-analysis. The diamond in the lower part of the graph depicts the pooled estimate along with 95% confidence intervals. Risk ratio (RR) was obtained using models with random effect (Mantel–Haenszel). Heterogeneity was assessed using the Q-test and quantified using the *I*^2^ value

In meta-analysis, FA was significantly protective against anastomotic leak (3 RCTs, 964 patients, RR: 0.67, 95% CI: 0.46 to 0.99, *I*^2^: 0%, *p* = 0.04) (Fig. 1). The risk difference in terms of incidence of AL was non-significantly decreased by 4 percentage units (95% CI: − 0.08 to 0, *I*^2^: 8%, *p* = 0.06) when using FA. This corresponds to a NNT of 25 patients.

### Quality ranking

The Cochrane Collaboration’s Risk of bias 2 tool identified some concerns of risk of bias among included RCTs (Table S3, Table S4). The concerns were mostly raised in domain 4 and caused by the absence of blinding of the outcome assessors.

### Certainty of evidence

GRADE assessment concluded to a moderate degree of certainty (Table S5). The certainty of evidence was not downgraded for the domains “inconsistency”, “indirectness” and “imprecision”. Of note, the domain “imprecision” was not downgraded, as (1) the 95% confidence interval of the effect estimate did not cross the clinical decision threshold, (2) the total number of patients included in our systematic review is bigger than the calculated sample size from included trials which were adequately powered, (3) FA has no serious adverse effects, minimal inconvenience and modest cost. The domain “risk of bias” was downgraded by 1 point as some concerns regarding the risk of bias were identified by the Cochrane Collaboration’s Risk of bias 2 tool.

## Discussion

In the present systematic review and meta-analysis, we showed that FA was protective against AL, with a pooled RR of 0.67 (95% CI: 0.46 to 0.99, *p* = 0.04, *I*^2^: 0%).

Interestingly, in the first RCT published in the field (in 2020), De Nardi et al. reported a tendency for FA to protect against AL in patients undergoing colorectal resection. Indeed, AL occurred in 11 patients (9%) in their control group and in six patients (5%) in their FA group, but this difference did not reach statistical significance (for a sample size of 240 patients) [15]. The same year, Alekseev et al. showed that FA significantly reduced the incidence of AL from 16.3% to 9.1% (*p* = 0.04). In subgroup analysis, the effect was only significant for lower anastomoses where the incidence of AL in the control group was highest. Also, the authors actively looked for potential AL by performing a water-soluble contrast enema or a pelvic CT within 30 days after the surgical procedure [16], which may explain the higher incidence of AL detected in the trial. Recently, Jafari et al. published the results of the ambitious PILLAR III trial. Briefly, the authors did not find any significant difference in terms of AL between FA and control procedure in 347 patients [17]. However, the trial was terminated before including the planned number of 450 patients due to low accrual rate, and therefore did not reach its minimal sample size calculated to show a potential difference between the intervention and the control groups.

Therefore, when looking at existing individual RCTs in the field, FA does not seem to reduce the incidence of AL in colorectal surgery, and the results shown by existing systematic reviews and meta-analyses of observational studies [7–9] are not confirmed by high-quality evidence [15–17]. Interestingly, Keller and Hompes commented on the PILLAR III trial, highlighting its methodological limitations, but also pointing out that its early termination did not allow it to reach its expected sample size, therefore preventing any definitive conclusion regarding the effectiveness of FA [21]. We believe that our meta-analysis supports the opinion of Keller and Hompes. Indeed, in pooled analysis, we showed that FA was protective against AL, with a RR of 0.67 (95% CI: 0.46 to 0.99, *p* = 0.04) and a low heterogeneity (*I*^2^: 0%). Therefore, we believe that the effect of FA on AL exists, but is of small magnitude. For instance, when increasing the sample sizes of existing RCTs using meta-analytic tools, the effect of AL became significant with low heterogeneity among trials. Further, the RD between FA and control was small (4 percentage units, despite being non-significant). This means that FA may potentially reduce the incidence of AL by 4 percentage units (from the control group to the intervention group), but also that individual trials were probably underpowered to show a potential effect of the technique due to the small magnitude of this effect (with an estimated NNT of 25). Moreover, the effect of FA on the prevention of AL was demonstrated by Alekseev et al. to be more pronounced for lower anastomoses, where the risk of AL is higher. Considering this, future RCTs in the field should probably target patients at higher risk for AL to be able to show a significant effect with a lower sample size.

We would also like to point out the existing heterogeneity in terms of methods used to perform FA in colorectal surgery, as we have previously reported [22]. The authors of included RCTs performed FA using different volumes of ICG and at different steps of the surgical procedures. Moreover, the interpretation of the perfusion of the bowel using FA was depending on subjective criteria and not on well-defined fluorescence criteria. In this regard, Lütken et al. identified time-to-peak of fluorescence, fluorescence slope and t_1/2_max, but not maximum intensity of fluorescence, as predictors of AL [23]. Therefore, we believe that future RCTs in the field should report fluorescence curves and precisely define fluorescence criteria used during surgical procedures to decide on the level of bowel division.

The strength of our meta-analysis is the following. First, existing systematic reviews and meta-analyses in the field have shown an encouraging effect of FA, but have been restricted to observational studies or non-randomised prospective studies. The effect of FA initially shown was unfortunately not confirmed by the most recent PILLAR III trial, and some teams have called to discontinue the intervention. However, the release of the PILLAR III trial has also allowed us to cumulate enough RCTs to perform the first systematic review and meta-analysis of RCTs in the field. We believe that this represents a major strength of our article, as the magnitude of effect of the intervention (FA) in our pooled analysis is less important than the effect usually reported by meta-analyses of non-randomised studies. This may be explained by an eventual publication bias, which should be explored by meta-analyses of observational studies and, if present, corrected for by a trim-and-fill analysis. However, the effect is still present and statistically significant, because our pooled analysis allowed to increase the sample size of existing RCTs, which were probably underpowered to show a significant effect.

The limitations of our systematic review and meta-analysis are the following: First, the low number of RCTs in the field, which did not allow performing of subgroup analysis, notably regarding the distance of the anastomosis from the anal verge, but also did not reach significance when assessing RD between FA and control patients; second, the heterogeneity in methods of performing FA and in assessing the incidence of AL; and third, reporting alternative outcomes other than AL would allow a more complete meta-analysis of RCTs and performing a reliable cost–benefit analysis of FA, which is still awaited.

To conclude, although not every individual RCT in the field has shown potential benefit of FA on AL, we showed that meta-analysis of these RCTs demonstrated that FA did protect against AL in colorectal surgery with a moderate certainty of evidence. Therefore, the results of the ongoing RCTs (IntAct:ISCRN 13334746, REC4T:NCT04637061, NCT01419860, ICG-COLORAL:NCT03602677 and others) are awaited with great interest before reaching definitive conclusions, in order to confirm the estimate of the effect by increasing the sample size of high-quality available evidence. Considering the potential small magnitude of effect of FA (which is estimated to be of 4 percentage units), we also advise that future RCTs include a cost–benefit analysis of the technique and better define the at-risk subpopulation who may benefit from FA.

## Supplementary Information

Below is the link to the electronic supplementary material.

Supplementary file1 (TIFF 6314 kb) PRISMA inclusion flowchart

Supplementary file2 (DOCX 13 kb) Literature search strategy

Supplementary file3 (DOCX 15 kb) Methods for fluorescence angiography

Supplementary file4 (DOCX 14 kb) Cochrane evaluation tool for risk of bias of included studies

Supplementary file5 (DOCX 60 kb) GRADE assessment

Supplementary file6 (DOCX 14 kb)

## Abbreviations

AL: Anastomotic leak
FA: Fluorescence angiography
RCT: Randomised controlled trial
