# Supplementary figures and images for: Fluorescence angiography likely protects against anastomotic leak in colorectal surgery: a systematic review and meta-analysis of randomised controlled trials
Source: Surg Endosc. 2022 May 4;36(10):7775–80. doi: 10.1007/s00464-022-09255-1 (PMC9485176; doi:10.1007/s00464-022-09255-1)

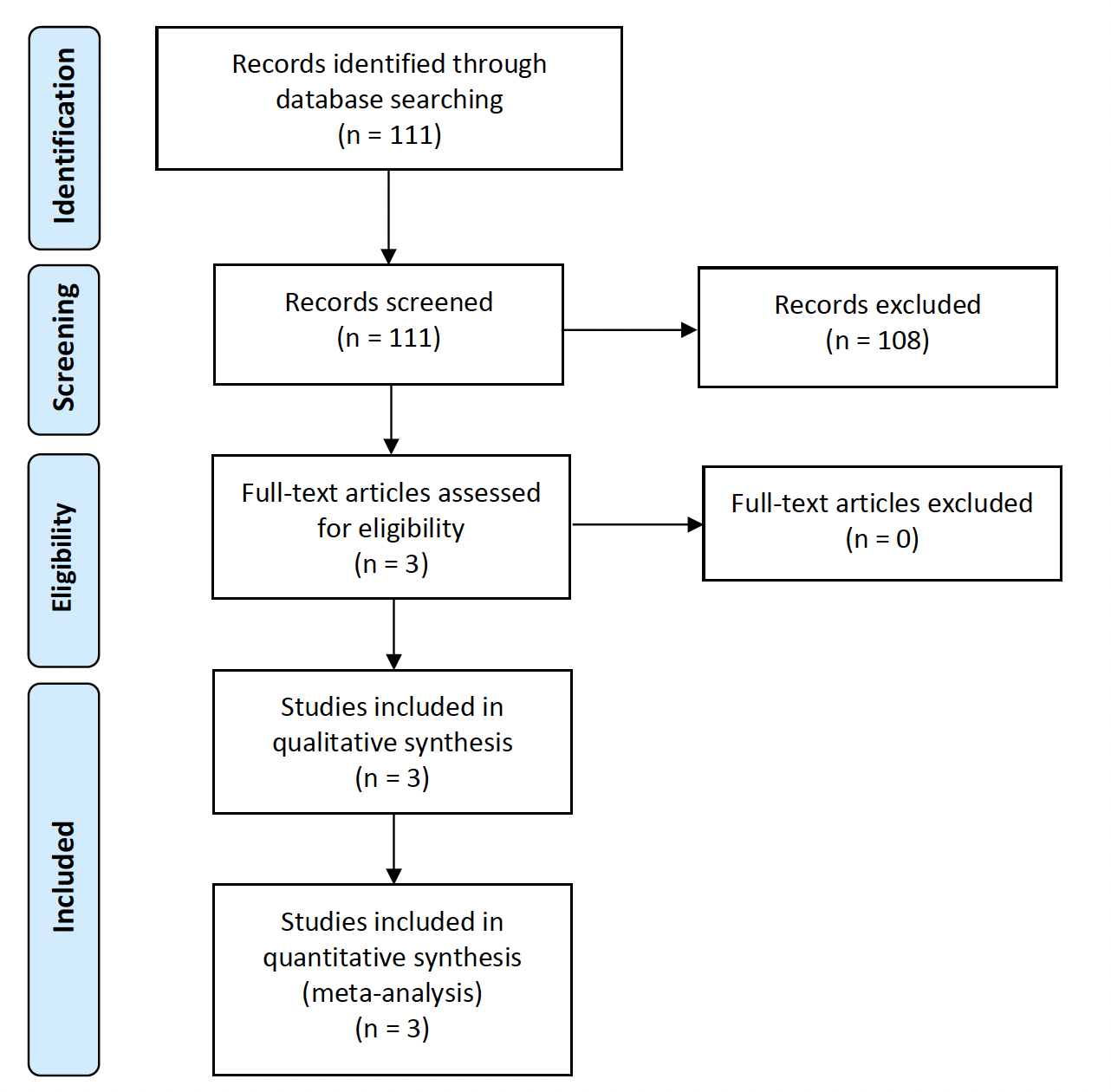

Supplement: Supplementary file 1 — Supplementary file1 (TIFF 6314 kb) PRISMA inclusion flowchart [file 464_2022_9255_MOESM1_ESM.tiff]
