# Supplementary material for: Fluorescence angiography likely protects against anastomotic leak in colorectal surgery: a systematic review and meta-analysis of randomised controlled trials
Source: Surg Endosc. 2022 May 4;36(10):7775–80. doi: 10.1007/s00464-022-09255-1 (PMC9485176; doi:10.1007/s00464-022-09255-1)
Supplement: Supplementary file 2 — Supplementary file2 (DOCX 13 kb) Literature search strategy [file 464_2022_9255_MOESM2_ESM.docx]

| **Database** | **Search build** | **Occurrences** |
| --- | --- | --- |
| MEDLINE | (angiography, fluorescence[MeSH Terms]) AND ((anastomotic leak[MeSH Terms]) OR (colorectal[Title/Abstract]) OR (anastom*[Title])) AND ((clinical trial[MeSH Terms]) OR (RCT[Title/Abstract]) OR (trial[Title/Abstract])) | 14 |
| EMBASE | 'fluorescence angiography':ti,ab,kw AND 'anastomotic leak':ti,ab,kw | 73 |
| COCHRANE CENTRAL | (*fluorescence angiography):ti,ab,kw AND (colorectal):ti,ab,kw AND (anastom*):ti,ab,kw | 23 |
| Other sources | - | 1 |
