# Supplementary material for: Fluorescence angiography likely protects against anastomotic leak in colorectal surgery: a systematic review and meta-analysis of randomised controlled trials
Source: Surg Endosc. 2022 May 4;36(10):7775–80. doi: 10.1007/s00464-022-09255-1 (PMC9485176; doi:10.1007/s00464-022-09255-1)
Supplement: Supplementary file 3 — Supplementary file3 (DOCX 15 kb) Methods for fluorescence angiography [file 464_2022_9255_MOESM3_ESM.docx]

| **Alekseev et al.** | |
| --- | --- |
| Intervention | |
| Dye | ICG 0.2mg/kg (PULSION Medical System) |
| Timing of injection | Before the division of the colon |
| Light source and detector | OPAL1 (KARL STORZ GmbH &Co) |
| Timing for analysis | 2-3 minutes after injection |
| Interpretation of good perfusion | Uniform distribution of fluorescence 2–3 min after ICG injection |
| Interpretation of bad perfusion | Uneven or absent distribution of fluorescence |
| Control | |
| Interpretation of perfusion | Visual assessment of the bowel |
| Intervention and control | |
| Additional control of the anastomosis | Air leak test |

| **De Nardi et al.** | |
| --- | --- |
| Intervention | |
| Dye | ICG 0.3mg/kg (PULSION Medical System) |
| Timing of injection | Before the division of the colon and after the completion of the anastomosis |
| Light source and detector | OPAL1 (KARL STORZ GmbH &Co) |
| Timing for analysis | At least 1 minute after injection and until a good perfusion signal is evident |
| Interpretation of good perfusion | Uniform distribution of fluorescence |
| Interpretation of bad perfusion | Non-uniform distribution of fluorescence to the chosen level of proximal colon resection or no fluorescence in the 10 cm proximal to the chosen level of colon resection |
| Control | |
| Interpretation of perfusion | Visual assessment of the bowel and assessment of perfusion of the marginal artery |
| Intervention and control | |
| Additional control of the anastomosis | Air leak test |

| **Jafari et al.** | |
| --- | --- |
| Intervention | |
| Dye | ICG 3.0+/-1.0ml of a 2.5-mg/ml solution |
| Timing of injection | After inferior mesenteric artery ligation and before bowel division, and after completion of the anastomosis through a transanal approach |
| Light source and detector | PINPOINT and/or SPY Elite near infrared range fluorescence imaging |
| Timing for analysis | - |
| Interpretation of good perfusion | Adequate: pale, dull, or faded green fluorescence; optimal: vivid, bright green fluorescence that entirely saturates the area of interest |
| Interpretation of bad perfusion | Absence of fluorescence or spotty and/or patchy areas of green fluorescence |
| Control | |
| Interpretation of perfusion | Standard practice |
| Intervention and control | |
| Additional control of the anastomosis | Air leak test |
