# Supplementary material for: Fluorescence angiography likely protects against anastomotic leak in colorectal surgery: a systematic review and meta-analysis of randomised controlled trials
Source: Surg Endosc. 2022 May 4;36(10):7775–80. doi: 10.1007/s00464-022-09255-1 (PMC9485176; doi:10.1007/s00464-022-09255-1)
Supplement: Supplementary file 4 — Supplementary file4 (DOCX 14 kb) Cochrane evaluation tool for risk of bias of included studies [file 464_2022_9255_MOESM4_ESM.docx]

| Study | Domain 1 | Domain 2 | Domain 3 | Domain 4 | Domain 5 | Risk of bias |
| --- | --- | --- | --- | --- | --- | --- |
| Alekseev et al. | Y-Y-N | Y-Y-PN-PN-PN | NI-N-PN | N-N-Y-PY-PN | N-PN-PN | Some concerns |
| De Nardi et al. | Y-Y-N | N-Y-NI | NI-N-PY-PN | N-N-Y-PY-PN | N-N-PN | Some concerns |
| Jafari et al. | Y-Y-N | Y-Y-NI-Y | NI-Y | N-N-Y-PY-PN | Y-N-N | Some concerns |
