# Supplementary material for: Fluorescence angiography likely protects against anastomotic leak in colorectal surgery: a systematic review and meta-analysis of randomised controlled trials
Source: Surg Endosc. 2022 May 4;36(10):7775–80. doi: 10.1007/s00464-022-09255-1 (PMC9485176; doi:10.1007/s00464-022-09255-1)
Supplement: Supplementary file 5 — Supplementary file5 (DOCX 60 kb) GRADE assessment [file 464_2022_9255_MOESM5_ESM.docx]

Revised Cochrane risk-of-bias tool for randomized trials (RoB 2)

TEMPLATE FOR COMPLETION

Edited by Julian PT Higgins, Jelena Savović, Matthew J Page, Jonathan AC Sterne
on behalf of the RoB2 Development Group

**Version of 22 August 2019**

The development of the RoB 2 tool was supported by the MRC Network of Hubs for Trials Methodology Research (MR/L004933/2- N61), with the support of the host MRC ConDuCT-II Hub (Collaboration and innovation for Difficult and Complex randomised controlled Trials In Invasive procedures - MR/K025643/1), by MRC research grant MR/M025209/1, and by a grant from The Cochrane Collaboration.


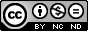


This work is licensed under a [Creative Commons Attribution-NonCommercial-NoDerivatives 4.0 International License](http://creativecommons.org/licenses/by-nc-nd/4.0/).

| **Study details**   \| **Reference** \| Alekseev et al. 2020 \| \| --- \| --- \|   **Study design**   \| X \| Individually-randomized parallel-group trial \| \| --- \| --- \| \| □ \| Cluster-randomized parallel-group trial \| \| □ \| Individually randomized cross-over (or other matched) trial \|   **For the purposes of this assessment, the interventions being compared are defined as**   \| Experimental: \| Fluorescence angiography \| Comparator: \| No fluorescence angiography \| \| --- \| --- \| --- \| --- \|  \| **Specify which outcome is being assessed for risk of bias** \| Anastomotic leak \| \| --- \| --- \|  \| **Specify the numerical result being assessed.** In case of multiple alternative analyses being presented, specify the numeric result (e.g. RR = 1.52 (95% CI 0.83 to 2.77) and/or a reference (e.g. to a table, figure or paragraph) that uniquely defines the result being assessed. \| Table 3 \| \| --- \| --- \|   **Is the review team’s aim for this result…?**   \| X \| to assess the effect of *assignment to intervention* (the ‘intention-to-treat’ effect) \| \| --- \| --- \| \| □ \| to assess the effect of *adhering to intervention* (the ‘per-protocol’ effect) \|   **If the aim is to assess the effect of *adhering to intervention***, select the deviations from intended intervention that should be addressed (at least one must be checked):  □ occurrence of non-protocol interventions  □ failures in implementing the intervention that could have affected the outcome  □ non-adherence to their assigned intervention by trial participants  **Which of the following sources were obtained to help inform the risk-of-bias assessment? (tick as many as apply)**  X Journal article(s) with results of the trial  □ Trial protocol  □ Statistical analysis plan (SAP)  X Non-commercial trial registry record (e.g. ClinicalTrials.gov record)  □ Company-owned trial registry record (e.g. GSK Clinical Study Register record)  □ “Grey literature” (e.g. unpublished thesis)  □ Conference abstract(s) about the trial  □ Regulatory document (e.g. Clinical Study Report, Drug Approval Package)  □ Research ethics application  □ Grant database summary (e.g. NIH RePORTER or Research Councils UK Gateway to Research)  □ Personal communication with trialist  □ Personal communication with the sponsor |
| --- | --- | --- | --- | --- | --- | --- | --- | --- | --- | --- | --- | --- | --- | --- | --- | --- | --- | --- | --- | --- |

## Risk of bias assessment

Responses underlined in green are potential markers for low risk of bias, and responses in red are potential markers for a risk of bias. Where questions relate only to sign posts to other questions, no formatting is used.

**Domain 1: Risk of bias arising from the randomization process**

| **Signalling questions** | **Comments** | **Response options** |
| --- | --- | --- |
| **1.1 Was the allocation sequence random?** | « Computer-generated randomization into two arms was  performed on the morning before surgery.”  « Computer-generated randomization lists, generated by the centre by means of envelopes, were allocated to each group. | Y |
| **1.2 Was the allocation sequence concealed until participants were enrolled and assigned to interventions?** |  | Y |
| **1.3 Did baseline differences between intervention groups suggest a problem with the randomization process?** | No baseline difference reported in Table 1. | N |
| **Risk-of-bias judgement-** | Low | Low |

Domain 2: Risk of bias due to deviations from the intended interventions (*effect of assignment to intervention*)

| **Signalling questions** | **Comments** | **Response options** |
| --- | --- | --- |
| **2.1. Were participants aware of their assigned intervention during the trial?** | “The investigators and patients were not blinded” | Y |
| **2.2. Were carers and people delivering the interventions aware of participants' assigned intervention during the trial?** |  | Y |
| **2.3. If Y/PY/NI to 2.1 or 2.2: Were there deviations from the intended intervention that arose because of the trial context?** | No deviation reported in the text and in the flowchart. | PN |
| **2.4 If Y/PY to 2.3: Were these deviations likely to have affected the outcome?** |  | NA |
| **2.5. If Y/PY/NI to 2.4: Were these deviations from intended intervention balanced between groups?** |  | NA |
| **2.6 Was an appropriate analysis used to estimate the effect of assignment to intervention?** | None reported. | PN |
| **2.7 If N/PN/NI to 2.6: Was there potential for a substantial impact (on the result) of the failure to analyse participants in the group to which they were randomized?** | Patients received the intervention to which they were allocated to. | PN |
| **Risk-of-bias judgement** | Some concerns | Some concerns |

Domain 3: Missing outcome data

| **Signalling questions** | **Comments** | **Response options** |
| --- | --- | --- |
| **3.1 Were data for this outcome available for all, or nearly all, participants randomized?** | Potential missing outcome values were not reported. | NI |
| **3.2 If N/PN/NI to 3.1: Is there evidence that the result was not biased by missing outcome data?** | No evidence. | N |
| **3.3 If N/PN to 3.2: Could missingness in the outcome depend on its true value?** | Potentially yes.  All patients with no anastomotic leak detected within 30 days underwent contrast enema or CT. Therefore, considering the number of investigators involved (incl. radiologists), it is unlikely that positive outcomes were deliberately missed. | PY |
| **3.4 If Y/PY/NI to 3.3: Is it likely that missingness in the outcome depended on its true value?** |  | PN |
| **Risk-of-bias judgement** | Some concerns | Some concerns |

Domain 4: Risk of bias in measurement of the outcome

| **Signalling questions** | **Comments** | **Response options** |
| --- | --- | --- |
| **4.1 Was the method of measuring the outcome inappropriate?** | The outcome was measured according to the International  Study Group of Rectal Cancer definition. Moreover, contrast enema or CT was performed at one month in patients without anastomotic leak. | N |
| **4.2 Could measurement or ascertainment of the outcome have differed between intervention groups?** | The methods used was appropriate and the same in the two groups. | N |
| **4.3 If N/PN/NI to 4.1 and 4.2: Were outcome assessors aware of the intervention received by study participants?** | “The investigators and patients were not blinded to the intervention details.” | Y |
| **4.4 If Y/PY/NI to 4.3: Could assessment of the outcome have been influenced by knowledge of intervention received?** | Potentially yes.  Outcome was assessed by a team of clinicians of different specialties, and involved imaging. | PY |
| **4.5 If Y/PY/NI to 4.4:** **Is it likely that assessment of the outcome was influenced by knowledge of intervention received?** |  | PN |
| **Risk-of-bias judgement** |  | Some concerns |

Domain 5: Risk of bias in selection of the reported result

| **Signalling questions** | **Comments** | **Response options** |
| --- | --- | --- |
| **5.1 Were the data that produced this result analysed in accordance with a pre-specified analysis plan that was finalized before unblinded outcome data were available for analysis?** | Primary outcome and its measurement were detailed in the study protocol NCT 03390517. However, in this protocol, measurement of the outcome was described as follows: “ After surgery on 7-8 POD patients are examined to two sides X-Ray proctography performed by introducing 100 ml of water-soluble liquid contrast material through the anus over the anastomotic line by a Foley catheter for diagnosis of anastomotic leakage. “ In the published manuscript: “If the postoperative period was entirely uneventful, a water-soluble contrast enema (Gastrografin  Bracco, Moscow, Russia) or pelvic CT was performed within 30 days after surgery with the aim of detecting a radiological leak.”  Moreover, there was no pre-specified data analysis plan. | N |
| **Is the numerical result being assessed likely to have been selected, on the basis of the results, from...** |  |  |
| **5.2. ... multiple eligible outcome measurements (e.g. scales, definitions, time points) within the outcome domain?** | Methods to measure the outcome differ between the clinicaltrials.gov protocol and the published manuscript (as described above). However, the timepoint mentioned in both documents is 30 days. The final analyses were performed on the outcome at 30 days. | PN |
| **5.3 ... multiple eligible analyses of the data?** | Probably no. | PN |
| **Risk-of-bias judgement** | Some concerns | Some concerns |

Revised Cochrane risk-of-bias tool for randomized trials (RoB 2)

| **Study details**   \| **Reference** \| De Nardi et al. 2020 \| \| --- \| --- \|   **Study design**   \| X \| Individually-randomized parallel-group trial \| \| --- \| --- \| \| □ \| Cluster-randomized parallel-group trial \| \| □ \| Individually randomized cross-over (or other matched) trial \|   **For the purposes of this assessment, the interventions being compared are defined as**   \| Experimental: \| Fluorescence angiography \| Comparator: \| No fluorescence angiography \| \| --- \| --- \| --- \| --- \|  \| **Specify which outcome is being assessed for risk of bias** \| Anastomotic leak \| \| --- \| --- \|  \| **Specify the numerical result being assessed.** In case of multiple alternative analyses being presented, specify the numeric result (e.g. RR = 1.52 (95% CI 0.83 to 2.77) and/or a reference (e.g. to a table, figure or paragraph) that uniquely defines the result being assessed. \|  \| \| --- \| --- \|   **Is the review team’s aim for this result…?**   \| □ \| to assess the effect of *assignment to intervention* (the ‘intention-to-treat’ effect) \| \| --- \| --- \| \| X \| to assess the effect of *adhering to intervention* (the ‘per-protocol’ effect) \|   **If the aim is to assess the effect of *adhering to intervention***, select the deviations from intended intervention that should be addressed (at least one must be checked):  □ occurrence of non-protocol interventions  □ failures in implementing the intervention that could have affected the outcome  □ non-adherence to their assigned intervention by trial participants  **Which of the following sources were obtained to help inform the risk-of-bias assessment? (tick as many as apply)**  X Journal article(s) with results of the trial  □ Trial protocol  □ Statistical analysis plan (SAP)  X Non-commercial trial registry record (e.g. ClinicalTrials.gov record)  □ Company-owned trial registry record (e.g. GSK Clinical Study Register record)  □ “Grey literature” (e.g. unpublished thesis)  □ Conference abstract(s) about the trial  □ Regulatory document (e.g. Clinical Study Report, Drug Approval Package)  □ Research ethics application  □ Grant database summary (e.g. NIH RePORTER or Research Councils UK Gateway to Research)  □ Personal communication with trialist  □ Personal communication with the sponsor |
| --- | --- | --- | --- | --- | --- | --- | --- | --- | --- | --- | --- | --- | --- | --- | --- | --- | --- | --- | --- | --- |

## Risk of bias assessment

Responses underlined in green are potential markers for low risk of bias, and responses in red are potential markers for a risk of bias. Where questions relate only to sign posts to other questions, no formatting is used.

**Domain 1: Risk of bias arising from the randomization process**

| **Signalling questions** | **Comments** | **Response options** |
| --- | --- | --- |
| **1.1 Was the allocation sequence random?** | “Patients meeting the inclusion criteria were randomized into two arms before the beginning of surgery by using a computerized random number generator list.”  « A sealed envelope containing the randomization arm was delivered after  the patient provided written informed consent, and it was opened before the surgical procedure began. » | Y |
| **1.2 Was the allocation sequence concealed until participants were enrolled and assigned to interventions?** |  | Y |
| **1.3 Did baseline differences between intervention groups suggest a problem with the randomization process?** | There is no baseline difference reported in Table 1 suggesting a problem with the randomization process. | N |
| **Risk-of-bias judgement-** | Low | Low |

Domain 2: Risk of bias due to deviations from the intended interventions (*effect of adhering to intervention*)

| **Signalling questions** | **Comments** | **Response options** |
| --- | --- | --- |
| **2.1. Were participants aware of their assigned intervention during the trial?** | “Patients were blinded to their treatment allocation.”  « This is a prospective, controlled (1:1), single-blinded, randomized, multicenter, superiority trial.” | N |
| **2.2. Were carers and people delivering the interventions aware of participants' assigned intervention during the trial?** |  | Y |
| **2.3. [If applicable:] If Y/PY/NI to 2.1 or 2.2: Were important non-protocol interventions balanced across intervention groups?** | Co-interventions were probably balanced between groups, but this was not documented in details. “A protective ileostomy was fashioned in all patients with previous long-course neoadjuvant radio-chemotherapy, with colo-anal anastomosis, intraoperative positive air leak test, and anastomosis located less than 5 cm from the anal verge.” | NI |
| **2.4. [If applicable:] Were there failures in implementing the intervention that could have affected the outcome?** | NA | NA |
| **2.5. [If applicable:] Was there non-adherence to the assigned intervention regimen that could have affected participants’ outcomes?** | NA | NA |
| **2.6. If N/PN/NI to 2.3, or Y/PY/NI to 2.4 or 2.5: Was an appropriate analysis used to estimate the effect of adhering to the intervention?** | NA | NA |
| **Risk-of-bias judgement** | Some concerns | Some concerns |

Domain 3: Missing outcome data

| **Signalling questions** | **Comments** | **Response options** |
| --- | --- | --- |
| **3.1 Were data for this outcome available for all, or nearly all, participants randomized?** | Potential missing outcome values were not reported. | NI |
| **3.2 If N/PN/NI to 3.1: Is there evidence that the result was not biased by missing outcome data?** | There is no such evidence. | N |
| **3.3 If N/PN to 3.2: Could missingness in the outcome depend on its true value?** | Potentially yes.  “The clinical suspicion of AL was confirmed by abdominal CT scan, contrast enema, endoscopy, or surgery.” Considering the number of investigators involved (incl. radiologists), it is unlikely that positive outcomes were deliberately missed. | PY |
| **3.4 If Y/PY/NI to 3.3: Is it likely that missingness in the outcome depended on its true value?** |  | PN |
| **Risk-of-bias judgement** | Some concerns | Some concerns |

Domain 4: Risk of bias in measurement of the outcome

| **Signalling questions** | **Comments** | **Response options** |
| --- | --- | --- |
| **4.1 Was the method of measuring the outcome inappropriate?** | “Patients were assessed during hospital stay and within 30 days of surgery based on clinical signs (fever, discharge from drainage, purulent discharge from the rectum, abdominal pain, deterioration of clinical condition, pelvic abscess, peritonitis) and laboratory parameters (leukocytosis or leukopenia, CRP and/or pro-calcitonin increase). The clinical suspicion of AL was confirmed by abdominal CT scan, contrast enema, endoscopy, or surgery.” The methods of measuring the outcome was appropriate. | N |
| **4.2 Could measurement or ascertainment of the outcome have differed between intervention groups?** | The methods used was appropriate and the same in the two groups. | N |
| **4.3 If N/PN/NI to 4.1 and 4.2: Were outcome assessors aware of the intervention received by study participants?** | Only patients were blinded. | Y |
| **4.4 If Y/PY/NI to 4.3: Could assessment of the outcome have been influenced by knowledge of intervention received?** | Potentially yes.  Outcome was assessed by a team of clinicians of different specialties, and involved imaging. | PY |
| **4.5 If Y/PY/NI to 4.4: Is it likely that assessment of the outcome was influenced by knowledge of intervention received?** |  | PN |
| **Risk-of-bias judgement** | Some concerns | Some concerns |

Domain 5: Risk of bias in selection of the reported result

| **Signalling questions** | **Comments** | **Response options** |
| --- | --- | --- |
| **5.1 Were the data that produced this result analysed in accordance with a pre-specified analysis plan that was finalized before unblinded outcome data were available for analysis?** | “The study was approved by the local ethic committees and registered at ClinicalTrials.gov (NCT02662946).”  However, there was no pre-specified data analysis plan. | N |
| **Is the numerical result being assessed likely to have been selected, on the basis of the results, from...** |  |  |
| **5.2. ... multiple eligible outcome measurements (e.g. scales, definitions, time points) within the outcome domain?** | The timepoint for measurement of the outcome (anastomotic leak) is indicated to be 30 days in both the clincialtrials.gov protocol and the published article. | N |
| **5.3 ... multiple eligible analyses of the data?** | Probably no. | PN |
| **Risk-of-bias judgement** | Some concerns | Some concerns |

Overall risk of bias

| **Risk-of-bias judgement** | Some concerns | Some concerns |
| --- | --- | --- |

| **Study details**   \| **Reference** \| Jafari et al. 2020 \| \| --- \| --- \|   **Study design**   \| X \| Individually-randomized parallel-group trial \| \| --- \| --- \| \| □ \| Cluster-randomized parallel-group trial \| \| □ \| Individually randomized cross-over (or other matched) trial \|   **For the purposes of this assessment, the interventions being compared are defined as**   \| Experimental: \| Fluorescence angiography \| Comparator: \| No fluorescence angiography \| \| --- \| --- \| --- \| --- \|  \| **Specify which outcome is being assessed for risk of bias** \| Anastomotic leak \| \| --- \| --- \|  \| **Specify the numerical result being assessed.** In case of multiple alternative analyses being presented, specify the numeric result (e.g. RR = 1.52 (95% CI 0.83 to 2.77) and/or a reference (e.g. to a table, figure or paragraph) that uniquely defines the result being assessed. \|  \| \| --- \| --- \|   **Is the review team’s aim for this result…?**   \| X \| to assess the effect of *assignment to intervention* (the ‘intention-to-treat’ effect) \| \| --- \| --- \| \| □ \| to assess the effect of *adhering to intervention* (the ‘per-protocol’ effect) \|   **If the aim is to assess the effect of *adhering to intervention***, select the deviations from intended intervention that should be addressed (at least one must be checked):  □ occurrence of non-protocol interventions  □ failures in implementing the intervention that could have affected the outcome  □ non-adherence to their assigned intervention by trial participants  **Which of the following sources were obtained to help inform the risk-of-bias assessment? (tick as many as apply)**  X Journal article(s) with results of the trial  X Trial protocol  □ Statistical analysis plan (SAP)  □ Non-commercial trial registry record (e.g. ClinicalTrials.gov record)  □ Company-owned trial registry record (e.g. GSK Clinical Study Register record)  □ “Grey literature” (e.g. unpublished thesis)  □ Conference abstract(s) about the trial  □ Regulatory document (e.g. Clinical Study Report, Drug Approval Package)  □ Research ethics application  □ Grant database summary (e.g. NIH RePORTER or Research Councils UK Gateway to Research)  □ Personal communication with trialist  □ Personal communication with the sponsor |
| --- | --- | --- | --- | --- | --- | --- | --- | --- | --- | --- | --- | --- | --- | --- | --- | --- | --- | --- | --- | --- |

## Risk of bias assessment

Responses underlined in green are potential markers for low risk of bias, and responses in red are potential markers for a risk of bias. Where questions relate only to sign posts to other questions, no formatting is used.

**Domain 1: Risk of bias arising from the randomization process**

| **Signalling questions** | **Comments** | **Response options** |
| --- | --- | --- |
| **1.1 Was the allocation sequence random?** | « The randomization schedules for all strata will be generated in advance by the contracted study statistician or designee using a computerized random number generator.”  « Randomization will be accomplished using a sequential numbered sealed envelope system. Due to stratification for prior neoadjuvant therapy, a box of envelopes will be supplied for each strata (one for subjects who have received prior neoadjuvant therapy and another for subjects who have not received prior neoadjuvant therapy). Envelope seals are broken and treatment assignment is made only after verification of proper informed consent execution and study eligibility. In order to prevent any attempts to subvert the randomization process, the subject’s initials, date of birth and date of randomization will be written on the randomization card. The randomization card will be signed by the study coordinator performing the randomization procedure and a second researcher who will witness the randomization procedure.” | Y |
| **1.2 Was the allocation sequence concealed until participants were enrolled and assigned to interventions?** |  | Y |
| **1.3 Did baseline differences between intervention groups suggest a problem with the randomization process?** | No baseline difference reported in Table 1. | N |
| **Risk-of-bias judgement-** | Low | Low |

Domain 2: Risk of bias due to deviations from the intended interventions (*effect of assignment to intervention*)

| **Signalling questions** | **Comments** | **Response options** |
| --- | --- | --- |
| **2.1. Were participants aware of their assigned intervention during the trial?** | “This is a multicenter randomized, controlled, unblinded, parallel study” | Y |
| **2.2. Were carers and people delivering the interventions aware of participants' assigned intervention during the trial?** |  | Y |
| **2.3. If Y/PY/NI to 2.1 or 2.2: Were there deviations from the intended intervention that arose because of the trial context?** | No deviation is reported in the text. No inclusion flowchart is available. | NI |
| **2.4 If Y/PY to 2.3: Were these deviations likely to have affected the outcome?** | NA | NA |
| **2.5. If Y/PY/NI to 2.4: Were these deviations from intended intervention balanced between groups?** | NA | NA |
| **2.6 Was an appropriate analysis used to estimate the effect of assignment to intervention?** | “The mITT analysis population includes all randomized subjects in whom a low anterior resection surgical procedure is initiated or at least one injection with ICG was performed. All subjects meeting these criteria are included in the mITT population, regardless of whether or not they received the planned open, or minimally invasive surgical intervention or NIR fluorescence imaging assessment using the PINPOINT or SPY Elite device.” | Y |
| **2.7 If N/PN/NI to 2.6: Was there potential for a substantial impact (on the result) of the failure to analyse participants in the group to which they were randomized?** | NA | NA |
| **Risk-of-bias judgement** | Some concerns | Some concerns |

Domain 3: Missing outcome data

| **Signalling questions** | **Comments** | **Response options** |
| --- | --- | --- |
| **3.1 Were data for this outcome available for all, or nearly all, participants randomized?** | The protocol specified what follows:  “ 10.8 Handling of Missing Data  Reasonable efforts will be made to obtain complete data for all patients; however, missing observations will inevitably occur due to patients lost to follow-up or noncompliance with required assessments. The reasons for missing data will be documented and evaluated (e.g. patient is deceased, lost to follow up, missed visit, etc.). In addition, the distribution of prognostic factors between patients with data and those without data will be examined to evaluate any potential sources of bias. Any missing observations will be described in detail and evaluated for assessment of possible bias. The planned sensitivity analyses for the primary outcome in the primary analysis population (including a tipping point analysis which addresses the worst case scenario in which treatment group subjects with missing observations are considered failures and control group subjects with missing observations are considered successful) is described in further detail in the Statistical Analysis Plan (SAP).”  However, the extent of missing data is not reported in the published article. | NI |
| **3.2 If N/PN/NI to 3.1: Is there evidence that the result was not biased by missing outcome data?** | “To confirm our primary efficacy interpretation, a tipping point sensitivity analysis was performed of our primary end point (AL). This analysis counted missing values for the perfusion group as no AL and counted missing values for the standard group as AL.” | Y |
| **3.3 If N/PN to 3.2: Could missingness in the outcome depend on its true value?** | NA  NA | NA |
| **3.4 If Y/PY/NI to 3.3: Is it likely that missingness in the outcome depended on its true value?** |  | NA |
| **Risk-of-bias judgement** | Low | Low |

Domain 4: Risk of bias in measurement of the outcome

| **Signalling questions** | **Comments** | **Response options** |
| --- | --- | --- |
| **4.1 Was the method of measuring the outcome inappropriate?** | “AL was defined as any evidence of endoluminal contents (air, fluid, GI contents, or contrast material) through the anastomosis as identified by imaging, drain output or at reoperation, or by endoscopic evidence of an anastomotic defect. Subjects who presented with a clinical suspicion of AL during the study who did not require urgent reoperation had a CT scan with oral and, if diverted or if necessary, rectal contrast to confirm. All scans were reviewed by an independent radiologist for confirmation.” | N |
| **4.2 Could measurement or ascertainment of the outcome have differed between intervention groups?** | The methods used was appropriate and the same in the two groups. | N |
| **4.3 If N/PN/NI to 4.1 and 4.2: Were outcome assessors aware of the intervention received by study participants?** | Yes. | Y |
| **4.4 If Y/PY/NI to 4.3: Could assessment of the outcome have been influenced by knowledge of intervention received?** | “The presence of infection or abscess thought to be related to the anastomosis was classified as an AL at the surgeon’s discretion even if it could not be definitively identified as visualized during an operation or by contrast extravasation.”  The number of investigators and centers involved make it unlikely. | PY |
| **4.5 If Y/PY/NI to 4.4: Is it likely that assessment of the outcome was influenced by knowledge of intervention received?** |  | PN |
| **Risk-of-bias judgement** | Some concerns | Some concerns |

Domain 5: Risk of bias in selection of the reported result

| **Signalling questions** | **Comments** | **Response options** |
| --- | --- | --- |
| **5.1 Were the data that produced this result analysed in accordance with a pre-specified analysis plan that was finalized before unblinded outcome data were available for analysis?** | Yes. Protocol number: PP PLR 03. | Y |
| **Is the numerical result being assessed likely to have been selected, on the basis of the results, from...** |  |  |
| **5.2. ... multiple eligible outcome measurements (e.g. scales, definitions, time points) within the outcome domain?** | No. | N |
| **5.3 ... multiple eligible analyses of the data?** | No. | N |
| **Risk-of-bias judgement** | Low | Low |

Overall risk of bias

| **Risk-of-bias judgement** | Some concerns | Some concerns |
| --- | --- | --- |


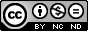


This work is licensed under a [Creative Commons Attribution-NonCommercial-NoDerivatives 4.0 International License](http://creativecommons.org/licenses/by-nc-nd/4.0/).
