# Supplementary material for: Fluorescence angiography likely protects against anastomotic leak in colorectal surgery: a systematic review and meta-analysis of randomised controlled trials
Source: Surg Endosc. 2022 May 4;36(10):7775–80. doi: 10.1007/s00464-022-09255-1 (PMC9485176; doi:10.1007/s00464-022-09255-1)
Supplement: Supplementary file 6 — Supplementary file6 (DOCX 14 kb) [file 464_2022_9255_MOESM6_ESM.docx]

| **Certainty assessment** | | | | | | | **№ of patients** | | **Effect** | | **Certainty** | **Importance** |
| --- | --- | --- | --- | --- | --- | --- | --- | --- | --- | --- | --- | --- |
| **№ of studies** | **Study design** | **Risk of bias** | **Inconsistency** | **Indirectness** | **Imprecision** | **Other considerations** | **Fluorescence angiography** | **control** | **Relative (95% CI)** | **Absolute (95% CI)** |  |  |
| 3 | randomised trials | serious | not serious | not serious | not serious | none | 39/483 (8.1%) | 58/481 (12.1%) | **RR 0.67** (0.46 to 0.99) | **40 fewer per 1 000** (from 65 fewer to 1 fewer) | ⨁⨁⨁ Moderate | CRITICAL |

**CI:** confidence interval; **RR:** risk ratio
